# Does size really matter? A multisite study assessing the latent structure of the proposed ICD-11 and DSM-5 diagnostic criteria for PTSD

**DOI:** 10.1080/20008198.2017.1398002

**Published:** 2017-11-13

**Authors:** Maj Hansen, Philip Hyland, Karen-Inge Karstoft, Henrik B. Vaegter, Rikke H. Bramsen, Anni B. S. Nielsen, Cherie Armour, Søren B. Andersen, Mette Terp Høybye, Simone Kongshøj Larsen, Tonny E. Andersen

**Affiliations:** ^a^ ThRIVE, Department of Psychology, University of Southern Denmark, Odense M, Denmark; ^b^ School of Business, National College of Ireland, IFSC, Dublin 1, Ireland; ^c^ Centre for Global Health, Trinity College Dublin, Dublin 2, Ireland; ^d^ Research and Knowledge Centre, The Danish Veteran Center, Ringsted, Denmark; ^e^ Pain Research Group, Pain Center South, Odense University Hospital, Odense C, Denmark; ^f^ Psychology Research Institute, Ulster University, Coleraine, Northern Ireland; ^g^ Interdisciplinary Research Unit, Elective Surgery Center, Silkeborg Regional Hospital, Silkeborg, Denmark; ^h^ Multidisciplinary Pain Center, Department of Anaesthesia and Intensive Care Medicine, Aalborg University Hospital, Aalborg, Denmark

**Keywords:** PTSD, DSM-5, ICD-11, CFA, diagnosis, TEPT, DSM-5, CIE-11, CFA, DIAGNÓSTICO, PTSD, DSM-5, ICD-11, CFA, 诊断, • What is the impact of using a large description of PTSD (i.e. the DSM-5) compared to a small description of PTSD (i.e. the ICD-11 proposal)? In other words, does the size of PTSD really matter? • The present multisite study compares diagnostic rates and model fit of competing DSM-5 and ICD-11 models of PTSD across pain patients, military personnel, and trauma-exposed university students (*N* = 4213). • The results show that the choice of diagnostic system may influence the estimated PTSD rates both qualitatively and quantitatively. Thus, the size of PTSD does matter. • The proposed ICD-11 three-factor model provided the best fit of the tested ICD-11 models across all samples. • The DSM-5 seven-factor Hybrid model and the DSM-5 six-factor Anhedonia model provided the best fit of the tested DSM-5 models.

## Abstract

**Background**: Researchers and clinicians within the field of trauma have to choose between different diagnostic descriptions of posttraumatic stress disorder (PTSD) in the DSM-5 and the proposed ICD-11. Several studies support different competing models of the PTSD structure according to both diagnostic systems; however, findings show that the choice of diagnostic systems can affect the estimated prevalence rates.

**Objectives**: The present study aimed to investigate the potential impact of using a large (i.e. the DSM-5) compared to a small (i.e. the ICD-11) diagnostic description of PTSD. In other words, does the size of PTSD really matter?

**Methods:** The aim was investigated by examining differences in diagnostic rates between the two diagnostic systems and independently examining the model fit of the competing DSM-5 and ICD-11 models of PTSD across three trauma samples: university students (*N* = 4213), chronic pain patients (*N* = 573), and military personnel (*N* = 118).

**Results**: Diagnostic rates of PTSD were significantly lower according to the proposed ICD-11 criteria in the university sample, but no significant differences were found for chronic pain patients and military personnel. The proposed ICD-11 three-factor model provided the best fit of the tested ICD-11 models across all samples, whereas the DSM-5 seven-factor Hybrid model provided the best fit in the university and pain samples, and the DSM-5 six-factor Anhedonia model provided the best fit in the military sample of the tested DSM-5 models.

**Conclusions**: The advantages and disadvantages of using a broad or narrow set of symptoms for PTSD can be debated, however, this study demonstrated that choice of diagnostic system may influence the estimated PTSD rates both qualitatively and quantitatively. In the current described diagnostic criteria only the ICD-11 model can reflect the configuration of symptoms satisfactorily. Thus, size does matter when assessing PTSD.

## Abstract (es)

**Planteamiento**: Los investigadores y clínicos del campo del trauma pronto decidirán entre dos descripciones diagnósticas diferentes del trastorno de estrés postraumático (TEPT) en el DSM-5 y la propuesta CIE-11. Varios estudios apoyan diferentes modelos en competencia sobre la estructura del TEPT en función de ambos sistemas de diagnóstico; sin embargo, los resultados demuestran que la elección de los sistemas de diagnóstico puede afectar las tasas de prevalencia estimadas.

**Objetivos**: y métodos. El presente estudio tenía como objetivo investigar el impacto potencial de usar una descripción del TEPT amplia (es decir, el DSM-5) en comparación con una pequeña (es decir, la CIE-11). En otras palabras, ¿el tamaño del TEPT importa realmente? El objetivo se investigó mediante el examen de las diferencias en las frecuencias de diagnóstico entre los dos sistemas de diagnóstico y examinando de forma independiente cómo se ajustaban los modelos en competencia para el TEPT del DSM-5 y la CIE-11 en tres muestras de trauma: estudiantes universitarios (*N* = 4213), pacientes con dolor crónico (*N* = 573) y personal militar (*N* = 118).

**Resultados**: Las tasas diagnósticas del TEPT fueron significativamente más bajas según los criterios de la propuesta CIE-11 en la muestra universitaria, pero no se encontraron diferencias significativas para los pacientes con dolor crónico y el personal militar. El modelo de tres factores propuesto por la CIE-11 proporcionó el mejor ajuste de los modelos de la CIE-11 que fueron probados en todas las muestras. En cambio, el modelo híbrido de siete factores del DSM-5 proporcionó el mejor ajuste en las muestras universitaria y del dolor, y el modelo de Anhedonia de seis factores del DSM-5 en la muestra militar de los modelos probados del DSM-5.

**Conclusiones**: Se pueden debatir las ventajas y desventajas de utilizar un conjunto amplio o reducido de síntomas para el TEPT; sin embargo, este estudio demostró que la elección del sistema de diagnóstico puede influir en las tasas estimadas del TEPT, tanto cualitativa como cuantitativamente. Al mismo tiempo, parece que, dados los criterios diagnósticos descritos actualmente, solo el modelo de la CIE-11 puede reflejar satisfactoriamente la configuración de los síntomas. Por lo tanto, el tamaño importa cuando se evalúa el TEPT.

## Abstract (zh)

**背景：**在创伤领域的研究者和临床工作者很快要在DSM-5和ICD-11提案种对两个不同的创伤后应激障碍(PTSD)诊断描述做出选择。一些研究根据不同的诊断系统支持不同的PTSD症状结构的竞争模型，但是研究发现显示不同的诊断系统的选择会影响对发生率的估计。

**目标和方法**：本研究旨在探究使用大量（如DSM-5）或者少量（如ICD-11）PTSD症状描述的潜在影响。换言之，PTSD的症状量是否真的重要？我们考察在两个不同诊断系统里的诊断率的差别，并分别在三个创伤样本里考察DSM-5和ICD-11 的PTSD竞争模型的拟合性：大学生样本（*N*=4213），长期疼痛病人（*N*=573）和军人样本（*N*=118）。

**结果：**在大学生样本里根据ICD-11提案的PTSD的诊断率显著更低，但是对长期疼痛病人和军人样本没有显著差别。ICD-11提案的三因子模型跨样本拟合最好，但DSM-5七因子混合模型在大学生和疼痛样本中拟合最好，DSM-5 六因子快感缺失模型在军人样本中拟合最好。

**结论：**使用一个广泛或者狭窄的PTSD症状集的优劣是可以争论的，但是本研究显示对诊断体系的选择可以从质性和量化角度影响对PTSD患病率的估计。同时，在目前的诊断标准中只有ICD-11模型可以令人满意地反映症状结果。因此在评估PTSD时，症状数量是重要的。

Clinicians and researchers are increasingly aware of the difficult decision facing them within the next years; deciding between the use of two rather different descriptions of posttraumatic stress disorder (PTSD; Hansen, Hyland, Armour, Shevlin, & Elklit, 2015). On the one hand, the *Diagnostic and Statistical Manual of Mental Disorders* 5th edition (DSM-5; American Psychiatric Association, 2013) describes PTSD as a diagnosis comprised of 20 symptoms belonging to four symptom clusters: intrusion, avoidance, negative alternations in cognitions and mood, and alternations in arousal and reactivity. On the other hand, the World Health Organization’s proposed 11th revision of the International Classification of Diseases (ICD-11) set for release in 2018 describes PTSD as comprised of only six symptoms belonging to three symptom clusters: re-experiencing, avoidance of traumatic reminders, and a sense of current threat manifested by excessive hypervigilance or enhanced startle reaction (Maercker et al., 2013). Thus, a key question is, does the size of PTSD matter? In other words, what impact does it have to use the large description of PTSD (i.e. the DSM-5) compared to the small description of PTSD (i.e. the ICD-11 proposal)?

Ultimately, different diagnostic systems may result in a PTSD diagnosis according to one system but not the other and vice versa. Since diagnostic criteria are used to guide clinical work in relation to screening and treatment as well as to inform the clinical understanding of the disorder (Elhai & Palmieri, 2011), it is crucial to identify a precise configuration of PTSD. Thus, the important question to ask here is, how are PTSD symptoms best described? The question does not have a straightforward answer as both diagnostic systems have been criticized for either being too complicated and thus difficult to use (i.e. the DSM-5; Maercker et al., 2013) or too simplistic, overlooking subjects with clinically significant PTSD symptoms (Wisco et al., 2016).

Since the release of the DSM-5, numerous studies have investigated the latent structure of DSM- 5 PTSD (Armour, 2015; for reviews see Armour, Müllerová, & Elhai, 2016; Hansen, Ross, & Armour, 2017). Several studies have supported the four-factor latent model of DSM-5 PTSD or alternative models using confirmatory factor analysis (CFA; Armour et al., 2016). The alternative supported models of the DSM-5 PTSD criteria include the four-factor Dysphoria model (Miller et al., 2013), the five-factor Dysphoric Arousal model (Elhai et al., 2011), the six-factor Anhedonia model (Liu et al., 2014), the six-factor Externalizing Behaviors model (Tsai et al., 2014), the six-factor Alternative Dysphoria model (Zelazny & Simms, 2015), and the seven-factor Hybrid model (Armour et al., 2015; for model symptom specifications see Table 1). A recent review of CFA studies comparing DSM-IV and DSM-5 models concluded that the DSM-5 model was a good representation of PTSD, but studies analysing five-, six-, and seven-factor models suggest a need of further alternations of the DSM-5 criteria for PTSD (Armour et al., 2016). The Anhedonia and the Hybrid models have shown promising results (see Armour et al., 2016; Soberón, Crespo, Gómez-Gutiérrez, Fernandez-Lansac, & Armour, 2017; Yang et al., 2017).

**Table 1.** Item mapping for the alternative DSM-5 PTSD factor models.

Symptoms	DSM-5 (four factors)	Dysphoria (four factors; Miller et al., 2013)	Dysphoric Arousal (five factors; Elhai et al., 2011)	Anhedonia (six factors; Liu et al., 2014)	External Behaviours (six factors; Tsai et al., 2014)	Alternative Dysphoria (six factors; Zelazny & Simms, 2015)	Hybrid (seven factors; Armour et al., 2015)
B1: Unwanted memories	I	I	I	I	I	I	I
B2: Disturbing dreams	I	I	I	I	I	I	I
B3: Reliving	I	I	I	I	I	I	I
B4: Feeling upset	I	I	I	I	I	I	I
B5: Physical reactions	I	I	I	I	I	I	I
C1: Internal avoidance	A	A	A	A	A	A	A
C2: External avoidance	A	A	A	A	A	A	A
D1: Amnesia	N	D	N	N	N	D	N
D2: Negative self-beliefs	N	D	N	N	N	D	N
D3: Self-blame	N	D	N	N	N	D	N
D4: Negative feelings	N	D	N	N	N	D	N
D5: Loss of interest	N	D	N	AN	N	AN	AN
D6: Distant	N	D	N	AN	N	AN	AN
D7: No positive feelings	N	D	N	AN	N	AN	AN
E1: Aggression	H	D	DA	DA	EB	EB	EB
E2: Risky behaviour	H	D	DA	DA	EB	EB	EB
E3: On guard	H	H	AA	AA	AA	AA	
E4: Easily startled	H	H	AA	AA	AA	AA	AA
E5: Concentration	H	D	DA	DA	DA	D	DA
E6: Sleep problems	H	D	DA	DA	DA	D	DA

Note: I = intrusions; A = avoidance; N = negative alternations in cognition and mood; H = hyperarousal; D = dysphoria; DA = dysphoric arousal; AA = anxious arousal; AN = anhedonia; EB = externalized behaviour.

Several studies have tested the latent structure of PTSD according to the proposed ICD-11 PTSD criteria (Cloitre, Garvert, Brewin, Bryant, & Maercker, 2013; Cloitre, Garvert, Weiss, Carlson, & Bryant, 2014; Elklit, Hyland, & Shevlin, 2014; Glück, Knefel, Tran, & Lueger-Schuster, 2016; Hansen et al., 2015; Hyland, Brewin, & Maercker, 2017a; Karatzias et al., 2016). Specifically, three different models of the ICD-11 PTSD criteria have been put forward and tested to varying extents, including a one-factor model (Glück et al., 2016), a two-factor-model (Forbes et al., 2015), and the original three-factor model (Hyland et al., 2017a; see Table 2 for symptom specifications). CFA studies testing only the three-factor ICD-11 model have generally shown excellent fit (Hansen et al., 2015; La Greca, Danzi, & Chan, 2017; Tay, Rees, Chen, Kareth, & Dilove, 2015). A few CFA studies have compared model fit of different models of ICD-11 PTSD within the same population (Forbes et al., 2015; Glück et al., 2016; Haravuori, Kiviuusu, Suomalainen, & Marttunen, 2016; Hyland et al., 2017a). Glück et al. (2016) found good fit in the three tested models with superior fit of the one-factor model of ICD-11. Forbes et al. (2015) and Haravuori et al. (2016) also reported good to excellent model fit in all the tested models with superior fit of the two-factor model. Hyland et al. (2017a) found that the three-factor model provided optimal fit.

**Table 2.** Item mapping for the alternative ICD-11 PTSD factor models.

Symptoms	ICD-11 Model	Two-factor Model (Forbes et al., 2015)	One-Factor Model (Glück et al., 2016)
Re1: Upsetting dreams	Re	Re-Av	PTSD
Re2: Re-experiencing in the here and now	Re	Re-Av	PTSD
Av1: Internal avoidance	Av	Re-Av	PTSD
Av2: External avoidance	Av	Re-Av	PTSD
Th1: On guard	Th	Th	PTSD
Th2: Easily startled	Th	Th	PTSD

Note: Re = Re-experiencing in the here and now; Av = Avoidance; Th = Threat.

Studies examining the consequences of choosing one system over the other are emerging. Some have examined the latent structure of the two diagnostic criteria in the same samples (Hansen et al., 2015; La Greca et al., 2017; Tay et al., 2015); others have compared the estimated prevalence rates (Hafstad, Thoresen, Wentzel-Larsen, Maercker, & Dyb, 2017; Hansen et al., 2015; Hyland et al., 2016; La Greca et al., 2017; O’Donnell et al., 2014; Stein et al., 2014; Wisco et al., 2016). According to Hansen et al. (2015), the three-factor ICD-11 model of PTSD provided excellent model fit in six out of seven samples with different traumatic events, whereas the investigated DSM-5 models performed poorly across all samples. In a similar vein, Tay et al. (2015) assessed the DSM-5 model and the ICD-11 PTSD model in West Papuan refugees (*N* = 230) and found poor fit of the DSM-5 model and good fit for the ICD-11 models. La Greca et al. (2017) assessed the DSM-5 model and the ICD-11 PTSD model in hurricane exposed children (*N* = 327) and found excellent fit of the ICD-11 model and adequate fit of the DSM-5 model. Additionally, several studies have compared the estimated PTSD DSM-5 and ICD-11 prevalence rates with mixed results. Some studies found significantly higher estimated PTSD rates with the DSM-5 PTSD compared to the ICD-11 criteria following different forms of traumatic exposure (e.g. victims of incest, veterans, whiplash patients; Hafstad et al., 2017; Hansen et al., 2015; Hyland et al., 2016; O’Donnell et al., 2014; Wisco et al., 2016). Other studies indicated no significant differences following different forms of traumatic exposure (e.g. sexual assault, physical assault, bereaved parents; Hafstad et al., 2017; Hansen et al., 2015; Stein et al., 2014). It is unclear from the La Greca et al. (2017) study if the elevated rate of DSM-5 PTSD (12.5%) compared to the ICD-11 (11.0%) was a significant difference, however diagnostic agreement was poor (45%).

Based on the above review it appears that different models of DSM-5 or ICD-11 are supported across different studies. The few studies testing both ICD-11 and DSM-5 models in the same populations appear to suggest that the ICD-11 based models may result in better fit than the DSM-5 based models. At the same time, there is also a tendency for ICD-11 to produce lower estimated PTSD prevalence rates than the DSM-5 criteria, but these differences may only be significant after certain types of traumatic exposure. Of note, it is important to stress that there is a lack of studies investigating the latent structure of ICD-11 and DSM-5 models in the same populations and using measurements developed specifically for both the ICD-11 and the DSM-5 criteria rather than just archival data and DSM-IV or DSM-5 based measurements to capture both disorders.

The present study therefore examined the latent structure of ICD-11 and DSM-5 models of PTSD in three different trauma populations – chronic non-cancer pain patients, trauma-exposed university students, and military personnel – using measurements specifically developed to assess the DSM-5 PTSD and the proposed ICD-11. First, the diagnostic rates of PTSD based on the DSM-5 and the proposed ICD-11 diagnostic algorithms were investigated and compared. Second, the statistical fit of the different DSM-5 and ICD-11 models (see Table 1 and Table 2 for model specifications) was assessed using CFA. It is important to stress that the models are not directly comparable per se as they use different numbers of indicators.

## 1. Materials and methods

### 1.1. Participants and procedures

Participants in the present study were drawn from three separate samples. All studies were granted the necessary ethical and legal approval according to Danish legislation.

#### 1.1.1. Sample 1: trauma-exposed university students (N = 5277)

Data was provided from a larger Danish university electronic questionnaire survey on interpersonal violence and physical and mental health conducted in November 2016 (*N =* 5277). A personal link to the questionnaire was sent out to the students’ email address. A total of 4213 (79.8%) indicated exposure to at least one traumatic event (64.4% female, *M* age = 24.92 years, *SD = *5.36, range 18–74) and were thus eligible for the present study. The most common traumatic events indicated as the most distressing events reported were ‘another’ trauma not listed (16.0%, *n* = 675), serious illness in others (14.4%, *n* = 605), death of relatives (13.5%, *n* = 569), and accidents (7.8%, *n* = 329).

#### 1.1.2. Sample 2: pain patients (N = 573)

Sample 2 comprised data from a larger ongoing web-based clinical registry (PainData) of patients with chronic non-malignant pain referred to assessment and treatment at one of three public multidisciplinary University Hospital Pain Clinics in Denmark. After referral to the clinic and before the initial consultation at the clinic, the patients were asked to fill out an electronic questionnaire sent via personal link to the patients’ official inbox, e-Boks (the channel that the Danish State and municipalities use to send official documents to citizens). The data provided for the present study were collected between February and December 2016 (35.6% female, *M* age = 48.60 years, *SD *= 14.86, range: 19–92). A total of 573 patients of 960 eligible participants (59.6%) reported exposure to a traumatic event. The most common types of traumas reported were accident (45.9%, *n* = 249), life-threatening illness (19.5%, *n* = 106), and sudden accidental death (19.0%, *n* = 103), whereas 7.7% (*n* = 42) were not specified.

#### 1.1.3. Sample 3: military personnel (N = 321)

Data were collected in 2014–2016 among Danish military personnel (*N = *321) deployed to the Middle East or Afghanistan in relatively stable deployment areas yet still war zones. A personal email with a link to the questionnaire was sent out to the personnel’s e-Boks. The respondents were first invited to fill out an electronic questionnaire. A reminder was sent where participants could freely choose between an electronic or an identical paper version of the questionnaire. In total, 61.4% were deployed with the army and 38.6% with the air force. In total, 118 indicated that they had been exposed to a traumatic event (91.5% male, *M* age = 35.85 years, *SD* = 10.28). The most common traumatic events were military traumatic events (e.g. enemy fire or improvised explosive devices: 66.9%, *n = *79), a civilian traumatic event after home coming (e.g. traffic accident or death of a family member: 9.3%, *n* = 11), and some other traumatic stressful events (e.g. not having a single traumatic event, but generally stressful deployment environment: 23.7%, *n* = 28).

### 1.2. Measures

Traumatic exposure was assessed as follows. In sample 1 a modified version of the Life Event Checklist-5 was used (LEC-5; Weathers et al., 2013a). The LEC-5 was modified to include childhood traumatic exposure explicitly as recommend by Hyland et al. (2017b) and modified according to the context of a Danish university sample (see Table 3 for specifications). Thus events that are not so common in Denmark and in the context of university students were excluded due to the limited amount of space in the questionnaire (e.g. severe human suffering, exposure to toxic substances). In sample 1, LEC-5 was used both to assess prior traumatic exposure as well as the index trauma for the filling out the PTSD measurements.

**Table 3.** Association between trauma exposure and DSM-5 and ICD-11 PTSD for the Danish University sample (*N = *4213).

	Total Exposed	DSM-5 PTSD	ICD-11 PTSD
Life Event	Valid % (*n*)	OR (95% CI)	OR (95% CI)
1. Natural disaster	15.9 (668)	1.14 (0.90, 1.45)	1.18 (0.87, 1.60)
2. Fire or explosion	9.5 (399)	1.54 (1.17, 2.02)**	1.51 (1.07, 2.14)*
3. Accident	29.3 (1236)	1.19 (0.98, 1.45)	1.39 (1.09, 1.78)*
4. Childhood neglect	8.9 (377)	5.13 (4.03, 6.52)***	4.46 (3.36, 5.94)***
5. Childhood physical assault	11.7 (494)	3.50 (2.80, 4.38)***	2.87 (2.17, 3.79)***
6. Childhood sexual abuse^a^	3.8 (161)	5.04 (3.59, 7.07)***	4.91 (3.36, 7.19)***
7. Other childhood sexual abuse^b^	12.2 (513)	2.86 (2.29, 3.58)***	3.50 (2.68, 4.57)***
8. Adult physical assault	18.2 (767)	2.05 (1.67, 2.53)***	2.24 (1.73, 2.89)***
9. Assault with a weapon	5.8 (246)	1.75 (1.25, 2.44)**	1.84 (1.21, 2.75)**
10. Adult sexual assault^c^	4.3 (183)	4.31 (3.12, 5.97)***	4.15 (2.85, 6.03)***
11. Other adult sexual assault^d^	12.5 (528)	2.58 (2.06, 3.24)***	2.65 (2.01, 3.50)***
12. Sickness	8.3 (351)	1.95 (1.48, 2.57)***	2.07 (1.48, 2.90)***
13. Violent death	6.1 (257)	2.10 (1.53, 2.87)***	2.04 (1.38, 3.01)***
14. Death of a relative	29.2 (1229)	1.83 (1.51, 2.20)***	1.74 (1.37, 2.22)***
15. Another trauma	43.4 (1828)	5.74 (4.63, 7.11)***	5.11 (3.87, 6.75)***

Note: CI = Confidence Interval. ^a^Sexual abuse in childhood (before age 18, for example being touched in a sexual way or being sexually abused by a parent or caregiver). ^b^Unwanted or unpleasant sexual experience (before age 18) other than scored in item 6. ^c^Sexual assault (from age 18, for example rape, attempted rape, made to perform any type of sexual act through force or threat of harm). ^d^Other unwanted or unpleasant sexual experience (from age 18). *p < .05, **p < .01, ***p < .001.

In sample 2, traumatic exposure was assessed dichotomously with the following fixed categories (natural disaster, accident [work or traffic], sexual assault, physical assault, life-threatening illness, sudden accidental death, other). Due to the limited amount of space in the questionnaire only the most prevalent types of traumatic exposure previously found in pain patients were assessed (Andersen, Andersen, Vakkala, & Elklit, 2012).

In sample 3, the respondents were asked to describe the most stressful event and report PTSD symptoms accordingly. The resulting open-ended responses were re-coded into four categories: military stressful, civilian stressful, another stressful, and no stressful event.

The PTSD checklist for DSM-5 (PCL-5; Weathers et al., 2013b) was administered to the participants in all three studies. The PCL-5 includes 20 items designed to measure the DSM-5 PTSD symptoms rated on a five-point Likert-type scale (0 = *not at all* to 4 = *extremely*) indicating how much a specific symptom has bothered the respondent in the past month. The scale can be used to generate a probable diagnosis of PTSD according to the DSM-5 criteria requiring participants to endorse at least one symptom of intrusion, one symptom of avoidance, two symptoms of negative alternations in cognitions and mood, and two symptoms of arousal, indicated by a score ≥ 2 (*moderately*). The PCL-5 has demonstrated acceptable reliability and validity (Bovin et al., 2016). Cronbach’s alpha (α) values for the three samples were satisfactory (total scale α values ranged from .94 to .97).

The International Trauma Questionnaire (ITQ; Cloitre, Roberts, Bisson, & Brewin, in preparation) was used to measure the proposed ICD-11 PTSD diagnosis. The ITQ is currently under development. Both applied versions of the ITQ measured PTSD with seven items. Re-experiencing is generally only measured with two symptoms as the third is an additional item designed to allow re-experiencing to be assessed in individuals without a clear memory of the event (Karatzias et al., 2016). Thus, only six items were used in the present study. The answers are rated on a five-point Likert-type scale (0 = *not at all* to 4 = *extremely*) indicating how much a specific symptom has bothered the respondents in the past month. The scale can be used to generate a probable diagnosis of PTSD according to the proposed ICD-11 criteria requiring the respondents to endorse at least one symptom of each in its three clusters, indicated by a score ≥ 2 (*moderately*). The ITQ has demonstrated initial acceptable reliability and validity (Karatzias et al., 2016). The α-values for the full scale across the three samples were satisfactory and ranged from .87 to .92.

### 1.3. Data analysis

The analytical strategy for the present study included two steps. First, ICD-11 and DSM-5 diagnostic rates were calculated within each sample, along with 95% confidence intervals (95% CI). These within-sample diagnostic rates were statistically compared using the Z-test. Additionally, diagnostic consistency across the classification systems was estimated using Cohen’s kappa statistic where a value greater than .61 indicates substantial agreement (Landis & Koch, 1977).

Second, the factorial validity of seven DSM-5 and three ICD-11 models of PTSD were investigated within an alternative model’s framework for each sample (see Table 1 and Table 2). CFA models were tested in Mplus 7.4 (Muthén & Muthén, 2013) using the robust maximum likelihood estimation (Yuan & Bentler, 2000). Traditional approaches to assessing model fit were followed whereby good model fit was indicated by a non-significant chi-square result, CFI and TLI values greater than .90 indicate adequate fit, and values greater than .95 indicate excellent fit; RMSEA and SRMR values less than .08 indicate adequate fit and values less than .05 indicate excellent fit. Model comparisons were conducted using the Bayesian Information Criterion (BIC) whereby the model with the lowest value is the best fitting.

## 2. Results

### 2.1. Diagnostic estimates

Among the university sample, the DSM-5 generated significantly higher rates of PTSD as compared to the ICD-11 (14.3% [95% CI 13.2–15.5%], *n =* 538 vs. 8.0% [7.2–8.9%], *n* = 302; Z = 8.64, SE = .01, *p* < .001). Some respondents met the diagnostic criteria for DSM-5 but not ICD-11 (*n* = 270), and a smaller number met criteria for ICD-11 but not DSM-5 (*n* = 32). Agreement between the two systems was moderate (Kappa = .60, SE = .02, *p* < .001).

No statistically significant difference between rates of DSM-5 and ICD-11 PTSD were observed within the chronic pain sample (16.6% [95% CI = 13.4–20.0%], *n* = 83 vs. 17.4% [14.4–20.8%], *n* = 87; Z = −0.34, SE = .02, *p* = .632). However, agreement across systems was moderate (Kappa = .60, SE = .05, *p* < .001): 26 respondents met DSM-5 criteria but not ICD-11, and respondents  30 met ICD-11 criteria but not DSM-5.

Within the military sample, rates of PTSD according to the DSM-5 were almost twice that of the ICD-11, but this difference was not statistically significant (9.6% [5.3–14.0%], *n* = 11 vs. 5.3% [95% CI = 2.6–8.8%], *n* = 6; Z = 1.24, SE = .03, *p* = .107). In this sample, five respondents met the DSM-5 criteria but did not meet the ICD-11 criteria, and there were no respondents who met diagnostic criteria for ICD-11 and did not meet the DSM-5 criteria. The diagnostic agreement was higher than the previous samples (Kappa = .68, SE = .13, *p* < .001).

Due to the significant differences in estimated prevalence rates across diagnostic systems in the university sample, we examined the risk of specific traumatic exposures for a probable PTSD diagnosis according to the ICD-11 and the DSM-5 in this sample. Furthermore, we examined the endorsement rates for each PTSD symptom cluster according to the two diagnostic systems for all three samples. As can be seen (Table 3), the associations between the different trauma types and the two diagnostic systems among the university sample were similar. Table 4 shows that endorsement of the DSM-5 symptom clusters was generally higher than the ICD-11 symptom clusters in the university sample, whereas only endorsement of DSM-5 intrusion was substantially higher compared to ICD-11 re-experiencing in the military sample. Finally, endorsement of avoidance was substantially higher for ICD-11 compared to the DSM-5 and the DSM-5 arousal was substantially higher than the ICD-11 sense of threat in the pain sample.

**Table 4.** Frequencies of endorsement of each DSM-5 and ICD-11 PTSD symptom cluster for each included Danish sample.

	University	Pain	Military
PTSD Symptom Cluster	Valid % (*n*)	Valid % (*n*)	Valid % (*n*)
DSM-5 Intrusions	41.6 (1,650)	31.6 (175)	22.2 (26)
DSM-5 Avoidance	34.4 (1,366)	23.0 (128)	19.0 (22)
DSM-5 NACM	35.0 (1,385)	47.3 (252)	17.9 (21)
DSM-5 Alterations in Arousal	25.8 (968)	61.4 (328)	17.1 (20)
ICD-11 Re-experiencing	15.1 (566)	31.6 (178)	6.9 (8)
ICD-11 Avoidance	28.5 (1,070)	34.9 (193)	19.1 (22)
ICD-11 Sense of Threat	23.6 (887)	32.6 (180)	16.2 (19)

### 2.2. Factorial validity of ICD-11 and DSM-5 PTSD

Table 5 shows the fit statistics for the alternative models of the ICD-11 symptoms of PTSD. In each sample, the three-factor model proposed for ICD-11 exhibited extremely close fit to the data, and was statistically superior to the one- and two-factor models. Across all samples, the ICD-11 model exhibited satisfactory model parameters results. All standardized factor loadings were > .60 and statistically significant (*p* < .001). Factor correlations were all significant (*p* < .001) and ranged from .69 to .97.

**Table 5.** Model fit statistics for the alternative models of ICD-11 PTSD symptoms in each included Danish sample.

	*χ^2^*	*df*	P	CFI	TLI	RMSEA (90% CI)	SRMR	BIC
*University Sample (*n* = 3966)*								
One-factor model	551.946	9	.000	.891	.819	.123 (.115–.132)	.049	55,169
Two-factor model	218.148	8	.000	.958	.921	.081 (.072–.091)	.031	54,467
**Three-factor model**	**11.287**	**6**	.**080**	.**999**	.**997**	.**015 (.000**–.**028)**	.**007**	**54,061**
*Pain Sample (*n* = 572)*								
One-factor model	113.159	9	.000	.888	.813	.142 (.120–.166)	.056	9081
Two-factor model	35.272	8	.000	.971	.945	.077 (.052–.104)	.036	8958
**Three-factor model**	**15.014**	**6**	.**020**	.**990**	.**976**	.**051 (.019**–.**084)**	.**024**	**8939**
*Military Sample (*n* = 118)*								
One-factor model	16.933	9	.049	.964	.939	.086 (.003–.149)	.032	1325
Two-factor model	17.100	8	.029	.958	.922	.098 (.030–.163)	.030	1328
**Three-factor model**	**6.175**	**6**	.**404**	.**999**	.**998**	.**016 (.000**–.**121)**	.**025**	**1315**

Note: Estimator = MLR; *χ2** = ***Chi-square Goodness of Fit statistic; *df* = degrees of freedom; P = Statistical significance; CFI = Comparative Fit Index; TLI = Tucker Lewis Index; RMSEA (90% CI) = Root-Mean-Square Error of Approximation with 90% confidence intervals; SRMR = Standardized Root-Mean Square Residual; BIC = Bayesian Information Criterion; Best fitting model in bold.

Table 6 reports the fit statistics for the alternative models of DSM-5 PTSD symptoms. Within each sample, the DSM-5 model of PTSD exhibited fit statistics that were generally at the border of acceptable fit and was amongst the poorest fitting of all models. In the university and pain samples, the seven-factor Hybrid model offered the best fit of the data, while in the military sample, the six-factor Anhedonia model offered the best fit.

**Table 6.** Model fit statistics for the alternative models of DSM-5 PTSD symptoms in each included Danish sample.

	*χ^2^*	*df*	P	CFI	TLI	RMSEA (90% CI)	SRMR	BIC
*University Sample (*n* = 3971)*								
DSM-5 model	2072.803	164	.000	.932	.922	.054 (.052–.056)	.033	187,915
Dysphoria model	2283.865	164	.000	.925	.913	.057 (.055–.059)	.036	188,330
Dysphoric arousal model	1729.446	160	.000	.944	.934	.050 (.048–.052)	.032	187,298
Anhedonia model	1023.913	155	.000	.969	.962	.038 (.035–.040)	.025	186,016
External Behaviors model	1574.885	155	.000	.950	.938	.048 (.046–.050)	.030	187,034
Alternative Dysphoria model	1682.662	155	.000	.946	.934	.050 (.048–.052)	.030	187,236
**Hybrid model**	**852.567**	**149**	.**000**	.**975**	.**968**	.**034 (.032**–.**037)**	.**022**	**185,738**
*Pain Sample (*n* = 573)*								
DSM-5 model	595.300	164	.000	.909	.894	.068 (.062–.074)	.055	27,446
Dysphoria model	502.445	164	.000	.928	.917	.060 (.054–.066)	.049	27,313
Dysphoric arousal model	464.578	160	.000	.935	.923	.058 (.052–.064)	.046	27,289
Anhedonia model	355.240	155	.000	.958	.948	.047 (.041–.054)	.034	27,164
External Behaviors model	425.704	155	.000	.943	.930	.055 (.049–.062)	.044	27,270
Alternative Dysphoria model	395.340	155	.000	.949	.938	.052 (.035–.049)	.038	27,220
**Hybrid model**	**298.495**	**149**	.**000**	.**968**	.**960**	.**042 (.038**–.**056)**	.**031**	**27,126**
*Military Sample (*n* = 117)*								
DSM-5 model	278.108	164	.000	.899	.883	.077 (.061–.092)	.049	4531
Dysphoria model	278.346	164	.000	.899	.883	.077 (.061–.093)	.049	4532
Dysphoric arousal model	275.345	160	.000	.898	.879	.078 (.063–.094)	.049	4543
**Anhedonia model**	**239.700**	**155**	.**000**	.**925**	.**908**	.**068 (.051**–.**085)**	.**047**	**4495**
External Behaviors model	268.851	155	.000	.900	.877	.079 (.063–.095)	.048	4553
Alternative Dysphoria model*	–	–	–	–	–	–	–	–
Hybrid model	231.968	149	.000	.927	.907	.069 (.051–.086)	.046	4509

Note: Estimator = MLR; *χ2** = ***Chi-square Goodness of Fit statistic; *df* = degrees of freedom; P = Statistical significance; CFI = Comparative Fit Index; TLI = Tucker Lewis Index; RMSEA (90% CI) = Root-Mean-Square Error of Approximation with 90% confidence intervals; SRMR = Standardized Root-Mean Square Residual; BIC = Bayesian Information Criterion; Best fitting model in bold.

*Model rejected due to a correlation > 1 between two latent factors (Externalizing behavior and Dysphoria).

## 3. Discussion

The present study compared the estimated PTSD prevalence rates according to the proposed ICD-11 PTSD criteria and the DSM-5 PTSD criteria, and examined the statistical fit of competing DSM-5 and ICD-11 PTSD models across three different trauma populations: university students, pain patients, and military personnel. The present study is the first multisite study assessing these aims using measurements developed specifically for the ICD-11 and the DSM-5 PTSD criteria, respectively.

We found no statistically significant differences in the estimated prevalence rate of PTSD in the pain and military samples. However, the PTSD rate was significantly more prevalent according to the DSM-5 than the ICD-11 in the university sample. This is somewhat congruent with existing research showing that the prevalence rates may only vary following some forms of traumatic exposure (Hafstad et al., 2017; Hansen et al., 2015; Hyland et al., 2016; O’Donnell et al., 2014; Stein et al., 2014; Wisco et al., 2016). At the same time, it is possible that the use of more accurate measurements of DSM-5 and ICD-11 PTSD reduce the difference in estimated prevalence rates. However, we did find a statistically significant difference in estimated PTSD prevalence rates in the university sample according to the proposed ICD-11 and the DSM-5, and the estimated kappa values in the pain and military samples were only around moderate to the low end of good. This suggests that although the number of respondents suffering from PTSD across the two diagnostic systems was similar the actual respondents behind these numbers were different. Thus, the use of the two diagnostic systems may result in both quantitatively and qualitatively different estimated prevalence rates. This is in accordance with previous studies, which have also found only low diagnostic agreement or moderate to good kappa values (Hafstad et al., 2017; Hansen et al., 2015; La Greca et al., 2017). Thus, in relation to diagnostic rates, it does indeed matter which diagnostic system is used. Additional analyses of the university sample showed that the risk of PTSD following different traumatic events were quite similar between the DSM-5 and the ICD-11 with the risk being highest following childhood traumatic exposure and sexual assault in adulthood. This may indicate that the prevalence rates may not deviate from each other in relation to the associated risk of PTSD following prior traumatic exposure.

Previous research has indicated that the differences in the estimated prevalence rates of the ICD-11 and the DSM-5 may be due to the re-experiencing criteria (Hafstad et al., 2017; Hyland et al., 2016; O’Donnell et al., 2014; Wisco et al., 2016) or arousal symptoms (Wisco et al., 2016), which is not surprising as this is where the two diagnostic systems are different. However, in the present study we found that especially the pain sample respondents showed a difference in the estimated rates of those meeting the avoidance criterion (i.e. DSM-5: 23.0% vs. ICD-11: 34.9%). Although the two avoidance items represent the same symptoms, the specific wordings in the questionnaires may result in different prevalence rates. Specifically, the two questionnaires use slightly different examples of internal avoidance; the DSM-5 states memories, thoughts, or feelings, whereas the ICD-11 TQ states thoughts, feelings, or physical sensations. The mentioning of physical sensations is likely to tap into the problems of separating PTSD from pain. Patients with chronic pain often experience the consequences of the traumatic event (i.e. chronic pain and disability) as important parts of the stressful experience, thus physical sensations such as pain may serve as a reminder of their loss and disability leading to distress that they tend to avoid. In this case, avoidance of internal reminders may be more related to loss and disability than to PTSD (Andersen et al., 2017).

Overall our results suggest there is a great deviation in who suffers from PTSD according to the two diagnostic systems, which may not be surprising as the two criteria were set forward on opposite grounds: ‘conceptualize PTSD broadly and provide full coverage of its clinical presentations’ (i.e. DSM-5; Weathers, 2017, p. 122) and conceptualize PTSD more narrowly and ‘simplify the diagnosis and direct clinicians’ attention to its core elements’ (i.e. ICD-11; Maercker et al., 2013, p. 1684). Consequently, the two diagnostic systems have very different visions of PTSD and what constitutes a clinically useful diagnosis (Wisco et al., 2016). The results of the present study showed that it matters which diagnostic system is used in relation to estimation of prevalence rates. Thus, the key question in relation to the present study is therefore which diagnostic system has the most precise configuration of PTSD that can be used to guide clinical work as well as to inform the clinical understandings and maintenance of the disorder?

The CFA results showed that the proposed ICD-11 three-factor model provided the best fit of the tested ICD-11 models across all samples. For the tested DSM-5 models, the Hybrid model provided the best fit in the university and pain samples, and the Anhedonia model in the military sample. Our results are in accordance with the Armour et al. (2016) review of CFA studies on DSM-IV and DSM-5 PTSD models, concluding that the DSM-5 factor structure may need further alternations from the DSM-5 four-factor model. Although the ICD-11 model and the DSM-5 models are not directly comparable per se due to the different number of indicators, our results clearly demonstrate that, when looking solely at the models proposed by the diagnostic criteria, the ICD-11 models appear to be better able to give a satisfactory reflection of the PTSD structure than the DSM-5 model. Hence, this may suggest that the prevalence rate of the proposed ICD-11 more precisely reflects the true rate of PTSD compared to the DSM-5. Indeed, a recent study by Shevlin, Hyland, Karatzias, Bisson, and Roberts (2017) shows that different latent models of DSM-5 PTSD symptoms produces different prevalence rates of PTSD in the same clinical population with lower rates associated with more complex models of the DSM-5 structure (i.e. six- and seven-factor models). As underlined by Shevlin et al. (2017), when testing latent models of PTSD, it is crucial to examine how psychometric models map onto diagnosis and the impact that a particular model can have on estimated prevalence rates of PTSD. In pain sample and university sample in the present study, the proportion of individuals meeting diagnostic status under the Hybrid model was significantly lower than the prevalence rates under the DSM-5 criteria (Pain: Hybrid: 8.9% (*n *= 50) vs. DSM-5: 16.6% (*n *= 83), Z = 3.68, SE = .02, *p* < .001; University: Hybrid: 7.0% (*n *= 275) vs. DSM-5: 14.3% (*n *= 538), Z = 10.42, SE = .01, *p* < .001). In the military sample, the use of the Anhedonia model diagnostic algorithm proposed by Shevlin et al. (2017) produced a non-significantly lower rate of PTSD of 8.5% (*n *= 10) compared to the estimated DSM-5 four-factor PTSD rates of 9.5% (*n *= 11, Z = .25, SE = .04, *p* = .40). Consistent with Shevlin et al. (2017) findings, application of the Hybrid model of PTSD led to substantially fewer people meeting diagnostic status. Furthermore, in the case of the university sample, where a significant difference existed between the two classification systems, adoption of a diagnostic algorithm consistent with the best fitting model of the structure of the DSM-based PTSD symptoms led to a prevalence estimate similar to that observed for ICD-11.

The results of the present study may have several implications for both clinical practice and for research. Although there was only a statistically significant difference between the estimated PTSD rates in the university sample, the kappa values indicated that the two systems did not capture the same individuals even when diagnostic rates were not significantly different. Thus, some participants had PTSD according to the ICD-11 and not the DSM-5 and vice versa. Depending on which diagnostic system reflects PTSD most accurately, our results indicate that a proportion of respondents may be over- or underdiagnosed with PTSD i.e. some respondents in need of treatment may not be offered any or the right treatment. In relation to the overall estimated differences in prevalence rates it is possible that the ICD-11 is underdiagnosing and, consequently, too few participants may be offered treatment; at the same time, too many respondents may be offered PTSD treatment that may not be needed if the DSM-5 is over-diagnosing (Hansen et al., 2015). Of note, the results also show that the actual individuals meeting the diagnostic criteria are not the same, so the choice of diagnostic system will greatly affect who is offered treatment. Thus, it is too simplified to say that ICD-11 is potentially underdiagnosing and the DSM-5 is potentially over-diagnosing. Although simpler diagnostic criteria may be attractive for both clinicians, researchers, and for patients for several reasons, it is crucial to state that simplification comes with a price. The results do suggest that a revision of the DSM-5 criteria is needed to ensure diagnostic precision to guide research, prevention, and treatment. Thus, far more research is still needed to determine which of the diagnostic system captures PTSD most correctly and for which reasons e.g. in multisite studies. Qualitative studies of how different patient groups interpret the questionnaires may be informative. For instance, comorbid pain may challenge the assessment of PTSD. This means that more knowledge is needed about how this patient group interprets questionnaire items. It is important to reach consensus about this as applying two such diverging systems will result in difficulties understanding the mechanisms underpinning PTSD and thus ultimately complicate research on preventive and treatment actions to be taken against PTSD.

The present study has some limitations. First, data for all samples were collected electronically, whereas the university study was an online survey only. The use of online surveys may explain the high level of traumatic exposure found within university samples, however, the use of electronic data collections is unlikely to have affected the results of the remaining two samples as these studies were still conducted in controlled settings. At the same time, there are also advantages associated with using electronic survey e.g. minimizing missing data. Second, PTSD symptoms were assessed using a self-report measure without the possibility of clinical verification of symptoms. Additionally, we were not able to assess the functional impairment criteria. It is important to stress that there are some unclarities associated with whether the reported index trauma (e.g. unspecified traumatic exposure and loss) meet the A criterion according to the DSM-5 across all three samples for minor proportions of the participants. Unfortunately, we do not have information to fully assess whether these events meet the A criterion in all participants. However, in relation to the university sample and military sample, the uncertainty is only in relation to the index trauma as all participants had been exposed to other traumas than loss, and unspecified traumas in the university sample and all the participants in the military sample were exposed to a war zone. Furthermore, although different types of traumatic exposures may be associated with different risks of PTSD (Conrad et al., 2017), a recent study shows that the latent structure of PTSD does not vary between participants who report a criterion A trauma and those who report a subthreshold stressor (Zelazny & Simms, 2015). Thus, the potential lack of A criterion endorsement may only have affected the estimated prevalence rates. However, if the prevalence rates have been affected then our results indicate that the ICD-11 and DSM-5 PTSD rates have been affected in similar ways as the risks found for ICD-11 and DSM-5 PTSD endorsement are very similar across traumatic exposures. Third, all sample sizes were large enough to conduct valid CFAs. However, it is possible that the difference between the estimated PTSD rates would have been significant in the military sample if the sample size had been bigger. Fourth, unfortunately it was not possible to test concurrent or discriminant validity in the present study as the included samples did not share these features. Finally, there is considerable overlap between the DSM-5 and the proposed ICD-11 PTSD, complex PTSD, and dissociative PTSD, which makes the comparison of the two diagnostic systems even more complicated. Although, the present study only concerns PTSD, future studies need to include the full range of trauma-related and comorbid disorders to obtain more comprehensive results.

## 4. Conclusions

The present study is the first multisite study to compare ICD-11 and DSM-5 prevalence rates and latent structure using disorder-specific measurement tools. This study demonstrated that the choice of diagnostic system will greatly influence the estimated PTSD prevalence rates both qualitatively and quantitatively which indicates the need for a revision of the factor structure of PTSD according to the DSM-5 but not the ICD-11. Indeed, the results provide empirical support for the construct of the proposed three-factor ICD-11 model for PTSD but not the four-factor DSM-5 PTSD model. Thus, it appears that in the current descriptions of the two diagnostic systems, the simple PTSD structure proposed by the ICD-11 may better reflect the configuration of symptoms, whereas the 20 DSM-5 symptoms appear to be better descripted as a six- or seven-factor model. Thus, the size of PTSD does matter in relation to the implications of using the large description of PTSD (i.e. the 20 DSM-5 symptoms) versus the small description of PTSD (i.e. the six ICD-11 symptoms). Hopefully, the future will bring more consensus on the configuration of PTSD to aid the prevention and treatment of victims of traumatic exposure.
